# Silver Thin-Film Plated Interconnected Metal Mesh Networks for Virus Detection and Prevention

**DOI:** 10.3390/mi16101177

**Published:** 2025-10-17

**Authors:** Tae Min Choi, Hwa Rim Lee, Sung Gyu Pyo

**Affiliations:** School of Integrative Engineering, Chung-Ang University, 84, Heukseok-ro, Dongjak-gu, Seoul 06974, Republic of Korea; c79411@gmail.com (T.M.C.); ghkfla0725@naver.com (H.R.L.)

**Keywords:** reusable masks, disease prevention, pollen allergens, electroless plating, virus inactivation

## Abstract

Many viruses, bacteria, and pollen that cause diseases such as COVID-19 are inhaled by humans as aerosols. Therefore, wearing a mask to block pathogen-containing aerosols is crucial for disease prevention. However, current masks have a drawback—residual bacteria on the mask surface can become aerosolized again, spreading infections. To address this, a reusable mask incorporating the antibacterial properties of silver particles should be developed to suppress the immune response to pathogens and pollen that contact the mask surface. This study analyzed protein surface changes in pollen shells following electroless silver plating on polypropylene (PP) filters of KF94 masks using microcurrent. Pore density increased from 7.94% before microcurrent application to 14.8% and 16.9%, depending on the duration of exposure. These results suggest that microcurrents alter pollen surfaces and affect the proteins in pollen shells that trigger hay fever, confirming the potential for preventing pollen allergies.

## 1. Introduction

The World Health Organization (WHO) declared COVID-19 a global pandemic on 11 March 2020, marking a critical moment in our history. With an estimated more than 776 million cases worldwide by September 2025 (see related links [1]), the disease’s high transmissibility continues to disrupt daily life despite decreased mortality due to vaccines. COVID-19 primarily spreads through aerosol droplets [2], and a return to pre-pandemic normalcy remains elusive, underscoring the urgent need for practical solutions.

mRNA COVID-19 vaccines trigger the body to produce antigens and antibodies, inducing both humoral and cell-mediated immunity. They are rapidly produced using genetic mRNA information, unlike traditional vaccines [3], which require the cultivation of pathogens. However, they have the limitation of short-lived immune response [4].

Pollen allergies, which are another threat to human beings, can cause asthma, rhinitis, conjunctivitis, and atopic dermatitis [5,6,7,8]. Global warming has increased pollen-producing species, worsening allergic reactions, especially in urbanized areas where pollen combines with combustion particles [6,9]. The most effective prevention for hay fever is the use of disposable masks or antihistamine treatments. However, antihistamines are less favored due to side effects like drowsiness, dizziness, and immunosuppression [10]. As a result, for protection against COVID-19 and [ollen allergies, physical blocking methods to prevent virus infections and reduce allergies have become increasingly important.

In 2011, concerns about the effectiveness of general masks against pollen were raised, as bioaerosols containing pollen, fungi, and viruses can cause reinfection [11,12]. HEPA filters can remove over 99.9% of fine dust [13,14], but their glass fiber components are harmful to humans and unsuitable for mask use [15,16,17]. Therefore, antibacterial treatments are necessary to eliminate bioaerosols on filters, prompting investigations into bacteria removal methods.

Also, Darnell et al. showed that heating SARS-CoV (Severe Acute Respiratory Syndrome Coronavirus) above 65 °C for more than 4 min destroyed its structure [18]. Various sterilization methods are now used on filters, such as heating, ultraviolet (UV) light, and washing with sterilizing liquids [19]. Heating the filter at 70 °C for 5 min can effectively inactivate viruses [20], and studies found that heating melt-blown nonwoven fabric (MNF) in masks at 85 °C for multiple cycles had minimal impact on filtration efficiency [21]. Additionally, UV-C radiation can reduce virus activity by damaging their DNA or RNA [22]. However, UV sterilization requires specialized equipment and the varying penetrating power of different UV wavelengths [23], making it impractical for daily use. Washing masks with sterilizing liquids, such as ethanol, is also effective but reduces the filter’s performance [24].

These methods are either inconvenient for daily use or reduce efficiency, leading to research on metallic coatings for masks. Silver, in particular, has shown high antimicrobial efficacy with minimal toxicity to humans [25,26,27,28,29,30]. Studies have demonstrated that silver-coated filters effectively eliminate bacteria through Ag^+^ ion release and surface interaction mechanisms [31,32], and a fiber generating weak microcurrents to suppress bacterial biofilm formation has also been developed [33]. This electroceutical fabric generates weak electric fields and has been found to inactivate viruses transferred from cells. From this perspective, Electroless plating (ELP), also known as auto-catalytic plating, is advantageous for depositing metals on non-conductive and low-conductivity polymers. It is non-destructive to the sample, especially important for delicate or highly sensitive materials. Moreover, the ELP method provides relatively uniform plating on fibrous materials, making it a reliable and convenient option for plating non-metals and polymers such as electrospun nanofibers and PP filters [34,35,36,37].

This study assesses the antibacterial properties of silver and explores the development of an electric mask capable of conducting microcurrents for sterilization. Silver thin films were deposited via ELP onto the inner PP filter of a KF94 mask. Additionally, surface structure modification of cottonwood pollen, a major allergen, was performed. The goal is to create an electric mask powered by a button-cell battery that is convenient and easy to use.

## 2. Materials and Methods

Ag deposition on the KF94 mask and polypropylene (PP) filter was carried out via ELP. The ELP process followed the classical Tollens-type reduction route [38,39,40,41].

Prior to plating, the polypropylene (PP) filter was sequentially cleaned with deionized (DI) water and ethanol for 3 min each at room temperature, followed by sensitization and activation to create catalytic sites for electroless deposition. The filter was immersed in a SnCl_2_–HCl solution (0.5625 g SnCl_2_ and 1 mL HCl in 250 mL DI water) for 3 min, then activated in a PdCl_2_–HCl solution (0.1875 g PdCl_2_ and 1 mL HCl in 250 mL DI water) for an additional 3 min under the same conditions.

Silver deposition was then performed by mixing AgNO_3_, NaOH, glucose, tartaric acid, ethanol, NH_4_OH, and DI water. Specifically, 1.75 g of silver nitrate (AgNO_3_) was dissolved in 60 mL of DI water to prepare the silver precursor solution. Separately, 0.75 g of sodium hydroxide (NaOH), 2.7 g of glucose, 0.24 g of tartaric acid, and 6 mL of ethanol were dissolved in 60 mL of DI water to form the alkaline reducing solution. The two solutions were then combined under continuous stirring at 25 °C, Subsequently, 10 mL of ammonium hydroxide (28–30 wt%) was added dropwise until the precipitate completely dissolved with 12 of total solution pH.

**Figure 1:**
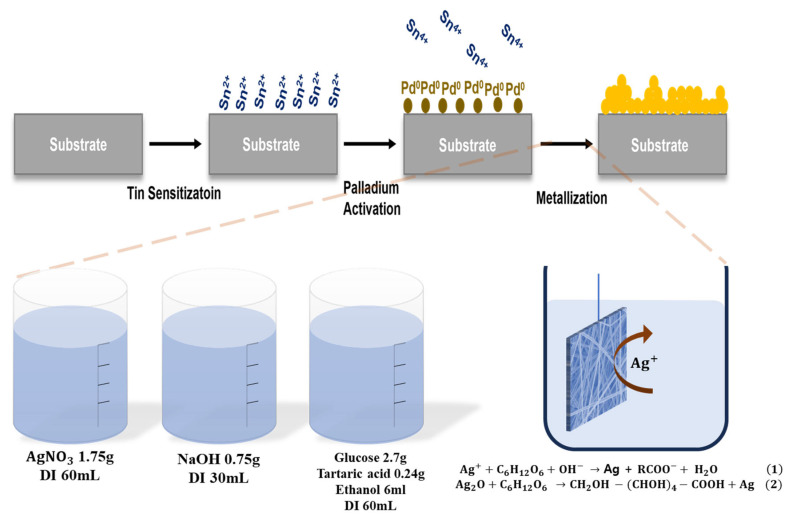
Experimental procedure for the electroless Ag plating process.

The KF94 mask filters were immersed in the resulting electroless plating bath and stirred at 25 °C. After plating, the samples were rinsed with DI water and dried at 70 °C under ambient conditions. Chemical reactions for Ag ELP are drawn in Figure 1.

The surface characteristics of the plated filter were analyzed using field emission scanning electron microscopy (FE-SEM, SIGMA 300, Carl Zeiss, Oberkochen, Deutschland), and the sheet resistance was measured with a 4-point probe to confirm conductivity.

A direct current power supply was connected to the plated filter, and a current of 1 mA was passed to observe the change caused by the microcurrent. Then, cottonwood pollen (Sigma Aldrich, St. Louis, MO, USA) was placed on the filter for 0–2 s, and the effect of the current on it was observed. Then, the diameter and density of the holes on the surface were calculated using FE-SEM and an image analysis program to evaluate the surface change.

## 3. Results and Discussion

### 3.1. PP Filter ELP

**Figure 2:**
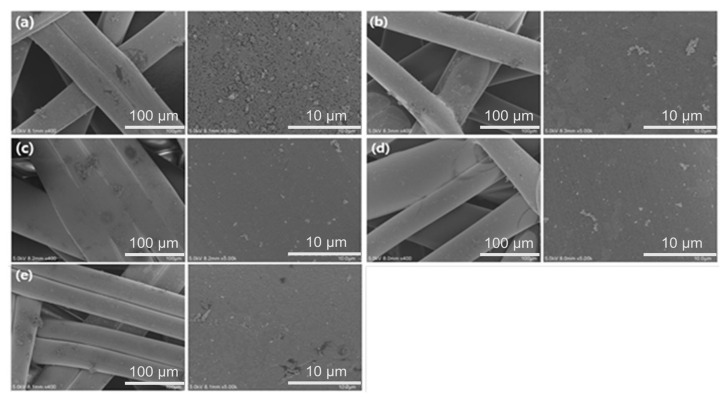
FE-SEM photographs: PP filter with Ag ELP for (**a**) 1 min, (**b**) 2 min, (**c**) 3 min, (**d**) 4 min, and (**e**) 5 min.

Different plating times (ranging from 1 to 5 min) were examined to identify the optimal conditions for achieving the highest conductivity and ensuring successful electroless plating on the PP filter. Figure 2 presents the FE-SEM images of the electroless silver-plated PP filter. Particles were formed on the plated filter after 1 min of the ELP process. After 2 min, silver was deposited in the form of a dense film.

**Figure 3:**
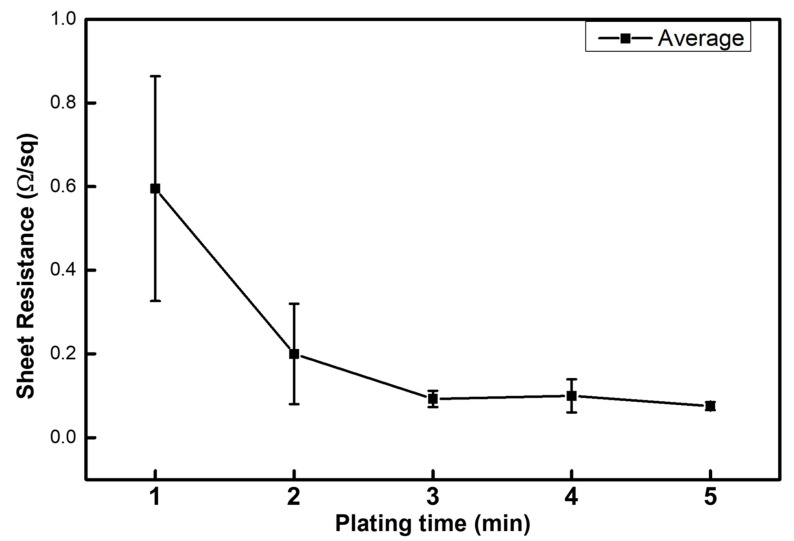
Sheet resistance change in PP + Ag filter according to the Ag electroless plating time.

Figure 3 shows a graph of sheet resistance based on the plating time of the mask filter. The fabricated PP filter + Ag filter had a sheet resistance of 0.6 Ω/sq after 1 min of electroless plating, which decreased to 0.2 Ω/sq after 2 min. After 3 min, it decreased to approximately 0.1 Ω/sq and stayed at a similar value for 5 min. The conductivity was confirmed for hay fever prevention and sterilization by applying current; the sheet resistance decreased as electroless plating progressed. The plating time was increased to further check the change in sheet resistance.

### 3.2. Variation in the Pollen Surface

**Figure 4:**
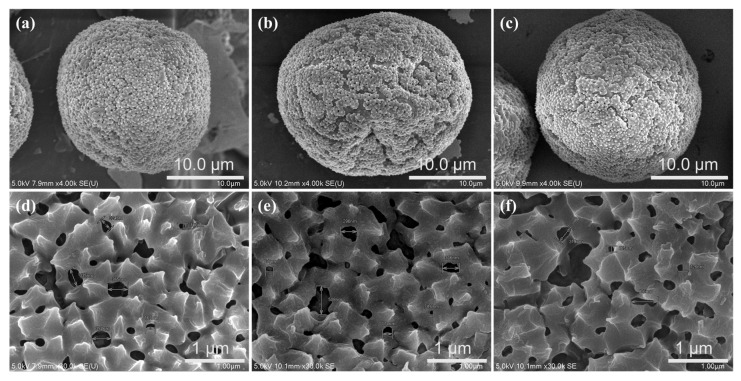
Variation in pollen surface on PP + Ag filter according to current application time: (**a**,**d**) before current application, (**b**,**e**) 1 s and (**c**,**f**) 2 s after application.

**Figure 5:**
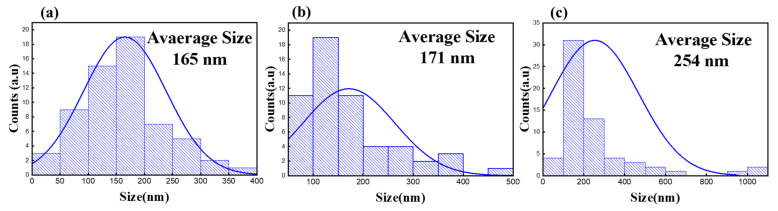
Variation in the hole diameter of the cottonwood pollen surface based on current application time, (**a**) no current, (**b**) 1 mA for 1 s, (**c**) 1 mA for 2 s.

FE-SEM and image analysis software were used to examine the changes in the cottonwood pollen surface following the application of an electric current. Two pollen samples were analyzed for each duration of current application. Figure 4 shows the FE-SEM images of the cottonwood pollen surfaces before and after the current application. The graph in Figure 5 presents the results of the hole diameter change analysis based on the current application time. The diameter of the surface holes increased with the application of current. Specifically, the hole area density in a 4 μm × 3 μm area of the pollen sample increased by 86% from before to 1 s after the current application. Consequently, the density of holes in the corresponding area increased from 7.94% before applying the current to 14.8% and 16.9%. The microcurrent-induced modification of pollen morphology may also be associated with conformational changes in surface or shell proteins that are responsible for allergenic reactions. Previous studies have reported that even low-intensity direct electric currents or weak electric fields can induce irreversible structural rearrangements in protein molecules [42,43]. These findings suggest that the weak current applied in this study could similarly affect pollen surface proteins, potentially reducing the antigen–antibody interaction responsible for allergic responses.

## 4. Conclusions

In this study, a silver-coated polypropylene (PP) filter for the KF94 mask was successfully fabricated via the electroless silver plating (ELP) method. This process provides a facile and scalable route to impart both electrical conductivity and antimicrobial functionality onto commercial polymer filters without high-temperature or vacuum-based deposition processes, unlike conventional sputtering or CVD methods. The sheet resistance of the silver-plated filter significantly decreased from 0.6 Ω/sq to 0.1 Ω/sq with increasing plating time, demonstrating stable current transport across the fibrous surface.

Furthermore, when a microcurrent of 1 mA was applied to the Ag-coated filter, surface modification of the adhered pollen particles was observed. FE-SEM analysis revealed an increase in pore density on the pollen surface from 7.94% to 14.8% and 16.9%, suggesting that the applied current induced morphological and possibly biochemical alterations of the pollen shell, which contains allergenic proteins. This behavior implies a potential new approach for in situ denaturation or structural modification of airborne allergens through controlled microcurrent exposure.

Compared with previous studies on antibacterial or antiviral filters that primarily rely on passive Ag ion release or photocatalytic reactions, the present ELP-based electrically active filter offers an additional electrochemical pathway for allergen deactivation under low-voltage conditions. This dual-function mechanism distinguishes it from conventional Ag-coated or electrospun functional filters.

Future work will focus on quantifying pollen adsorption and allergen reduction efficiency in simulated breathing environments, as well as evaluating differential pressure and adhesion characteristics to optimize comfort and filtration performance for real mask applications.

## Data Availability

Dataset available on request from the authors.
